# Migraine and psychiatric comorbidity: a review of clinical findings

**DOI:** 10.1007/s10194-010-0282-4

**Published:** 2011-01-06

**Authors:** Fabio Antonaci, Giuseppe Nappi, Federica Galli, Gian Camillo Manzoni, Paolo Calabresi, Alfredo Costa

**Affiliations:** 1 University Centre for Adaptive Disorders and Head pain (UCADH), Pavia, Italy; 2 Headache Medicine Centre, Policlinic of Monza, Monza, Italy; 3 Department of Child and Adolescent Neuropsychiatry, University La Sapienza, Rome, Italy; 4 Department of Neuroscience, Headache Centre, University of Parma, Parma, Italy; 5 Neurology Clinic, S. Maria della Misericordia Hospital, University of Perugia, Perugia, Italy; 6 National Institute of Neurology IRCCS C, Mondino Foundation Pavia, Pavia, Italy; 7 University Consortium for Adaptive Disorders and Head pain (UCADH), Via Mondino 2, Pavia, 27100 Italy

**Keywords:** Migraine, Comorbidity, Psychiatric disorders, Depression, Meta-analysis

## Abstract

Migraine is an extremely common disorder. The underlying mechanisms of this chronic illness interspersed with acute symptoms appear to be increasingly complex. An important aspect of migraine heterogeneity is comorbidity with other neurological diseases, cardiovascular disorders, and psychiatric illnesses. Depressive disorders are among the leading causes of disability worldwide according to WHO estimation. In this review, we have mainly considered the findings from general population studies and studies on clinical samples, in adults and children, focusing on the association between migraine and psychiatric disorders (axis I of the DSM), carried over after the first classification of IHS (1988). Though not easily comparable due to differences in methodology to reach diagnosis, general population studies generally indicate an increased risk of affective and anxiety disorders in patients with migraine, compared to non-migrainous subjects. There would also be a trend towards an association of migraine with bipolar disorder, but not with substance abuse/dependence. With respect to migraine subtypes, comorbidity mainly involves migraine with aura. Patients suffering from migraine, however, show a decreased risk of developing affective and anxiety disorders compared to patients with daily chronic headache. It would also appear that psychiatric disorders prevail in patients with chronic headache and substance use than in patients with simple migraine. The mechanisms underlying migraine psychiatric comorbidity are presently poorly understood, but this topic remains a priority for future research. Psychiatric comorbidity indeed affects migraine evolution, may lead to chronic substance use, and may change treatment strategies, eventually modifying the outcome of this important disorder.

## Introduction

Migraine is an extremely common disorder, characterized by the recurrence of painful and non-painful episodic phenomena and a variety of neurological manifestations. Nosographically, it is thus a chronic illness (migraine seen as a “disease”) interspersed with acute signs and symptoms (migraine seen as an “attack”). The mechanisms underlying migraine appear to be increasingly complicated, and the term complex disease is used to define the nature of the illness and to describe the whole breadth of the clinical and subclinical aspects that it encompasses [1, 2]. The wide heterogeneity of migraine accounts for the observation that the large population of migraine sufferers includes patients living an almost normal life and patients complaining of serious disability, i.e. facing social, affective and occupational limitations [3].

While genetic determinants are certainly at the basis of some (and probably all) clinical forms, the contribution of biological factors critically affects the clinical appearance of migraine. Recent findings in the field of neurogenetics have altered deeply our approach to migraine, emphasising the limits of the current diagnostic and nosographic system. With the current diagnostic criteria of International Headache Society (IHS) [4] allowing a better phenotypical characterisation of patients, the importance of the role of genetics in the mechanisms of migraine is increasing. In most cases, however, migraine occurs as multifactorial inherited character. Therefore, different genes or loci may interact with factors intrinsic to the individual (e.g. the hormone milieu) and/or with exogenous factors (e.g. psychosocial stressors related to the family or to the working environment, geoclimatic changes, food), generating different clinical forms of the disease [5].

Another important aspect of migraine heterogeneity, in close proximity to the field of genetic determinants, is the significant association between migraine and other neurological diseases (such as epilepsy, cerebrovascular disorders and stroke, mitochondrial diseases), cardiovascular disorders (arterial hypertension, mitral valve prolapse), and particularly psychiatric illnesses (anxiety, affective and personality disorders) [6]. This non-coincidental association of two or more diseases, referred to as comorbidity, may result from different mutations in the same gene (allelic disease) or mutations in genes located in neighbouring segments of the same chromosome.

A further aspect of migraine heterogeneity, extremely relevant to clinical research, is that the phenotypical expression of comorbidity may vary over time. This emerges upon simple observation of the natural history of migraine in the lifetime of different individuals. The phenotypical manifestations remain unchanged over the years in some patients, while in others the clinical picture becomes more complicated, and may include arterial hypertension (per se a risk factor for cerebrovascular accidents) and/or anxiety and mood disturbances. On the other hand, the presence of hypertension and psychiatric disorders often facilitates changes in the migraine pattern, resulting in forms of daily headache now referred to as chronic migraine [7].

It would therefore appear that the clinical-descriptive approach to the patient, demanded by the current diagnostic criteria, allows only a partial understanding of migraine, the nature of which certainly appears to be more complex and heterogeneous than previously thought.

## Migraine psychiatric comorbidity

The relationship between migraine and certain psychological features, such as a tendency toward perfectionism, neuroticism, repressed aggressivity and melancholic mood has been repeatedly reported for more than a century. Over the years, several data in the literature have been collected on anedoctical bases. Recently, with the development of new diagnostic criteria and statistical methodology, some of these observations have been confirmed [8, 9], but it is impossible to compare across all previous investigations due to differences in nosography and case definition. For instance, earlier than 2004, some nosologic entities, such as chronic migraine, which is frequently comorbid with psychiatric disorders, were not defined by diagnostic criteria.

Understanding the psychiatric correlates of migraine is critical for several reasons. Depressive disorders are among the leading causes of disability worldwide [10] and the WHO estimates that major depressive disorder will become the second leading cause of disease burden by the year 2020, second only to ischemic heart disease [11]. Migraine is a public health problem with an enormous impact on both the individual sufferer and on society. It is per se the most burdensome of the primary headache disorders [12, 13]. The presence of psychiatric conditions is a risk factor for transformation of migraine into a chronic form [7]. Furthermore, individuals with migraine and comorbid psychiatric disorders are greater health resources users than migraineurs without psychiatric conditions. Recognizing this comorbidity should therefore result in improved patient management, via first-line treatment targeted at both conditions.

In this review, we have mainly considered the studies carried over after the publication of the first classification of the IHS [14], in adults and children, focused on the association between migraine and psychiatric disorders, as defined on axis I of the Diagnostic and Statistical Manual of Mental Disorders (DSM) [15].

## General population studies

Since the first introduction of the IHS criteria [14], several studies of community-drawn samples have examined migraine psychiatric comorbidity. All cross-sectional investigations of psychiatric disorder prevalence in ‘migraine’ compared with ‘non-migraine’ samples found an increased risk of anxiety disorders, particularly panic disorder (PD) and phobias. As for other anxiety syndromes, one study [16] found an association between migraine and obsessive–compulsive disorder (OCD) as well as generalized anxiety disorder (GAD), but these associations were not replicated in subsequent investigations [17–19]. All these investigations were of high methodological quality (including rigorous sampling criteria in a community setting, use of structured interviews and application of DSM diagnostic criteria). The reasons for this discrepancy therefore remain uncertain.

Previous investigations of mood disorders have been highly consistent in reporting an increased prevalence of major depressive disorder (MDD) in patients with migraine [6]. The only one discrepant study [20] was characterized by several methodological differences and limitations that may account for the lack of any association between MDD and migraine. First, the patients studied were of considerably younger age and were not sex-matched by gender. Second, the sample was not enough representative of the general population or of clinical settings, since the study group included young adults selected for ‘high psychopathological risk’ as detected by a general symptom scale. Finally, the recruitment of the clinical cases was based on the use of a structured diagnostic interview (SPIKE) originally designed for epidemiological studies and not specifically for psychiatric diagnostic purposes. An increased risk of current depression in migraine patients was also reported in a community-based study [21]. This investigation included an assessment of quality of life, and the conclusions of the authors were that both migraine and depressive disturbances exert a significant but independent impact on this parameter. Furthermore, in another study in patients over 65 years, the risk of current depression was found to be increased in migraine patients compared to healthy controls [22] and headache appeared to be independently associated with depression in the elderly. However, the diagnostic instruments in this setting included scales not specifically intended for the assessment of DSM criteria. Further evidence of a high prevalence of depressive symptoms in adult to elderly patients with migraine was provided by a recent cross-sectional study [23].

In patients with migraine with aura, an association was early reported with suicide attempts, even after adjustment of data for major depression [16]. This observation is in line with later studies by the same group claiming that migraine with aura is characterized by psychiatric comorbidity more frequently than migraine without aura [24]. Interestingly, similar findings were recently obtained in a sample of adolescents aged 13–15 years. A higher frequency of suicidal ideation was observed in younger adolescents with migraine with aura or with high frequency of attacks, these associations being independent of depressive symptoms [25].

With regard to other forms of affective disorder, in one of the studies [16] only migraine with aura was found to be significantly associated with bipolar disorder (BD). Later observations did not confirm this association, but they did not distinguish between migraine subtypes [17]. Recently, a significant association between migraine and BD was reported by one study [18], with migraine subjects showing BD twice as often as those without migraine. Results were further confirmed by a subsequent, large study in a population-based sample [19].

In cross-sectional investigations, substance-related disorders have been examined in a few cases. An increased risk of alcohol and drug abuse was reported in migraine sufferers [16], but subsequent studies could not replicate this finding [20]. One possible explanation for such discrepancy may be related to the fact that BD and substance abuse are highly comorbid. However, more recent studies have shown again no association between migraine and drug, alcohol or substance abuse/dependence [18, 19].

The prospective analyses carried over in almost all the mentioned studies were also addressed to measure the risk of psychiatric disorders in subjects primarily suffering from migraine. For instance, the risk of panic disorder and phobia onset during the follow-up period was found to be greater for patients with migraine than for those without migraine [26]. However, in this study, no clear significant association was reported between migraine and affective disorders, at variance with other authors [28], possibly due to the different headache diagnostic criteria used. In a study following patients for up to 15 years, the presence of phobia at the basal evaluation was also shown to predict the subsequent occurrence of migraine [17]. Recently, Ratcliffe et al. [19] examined for the first time the relationship between physician-diagnosed migraine and multiple psychiatric disorders in a large, nationally representative sample. Important points overcoming the limitations of previous studies were the use of a standardized interview (Composite International Diagnostic Interview, CIDI) and the fact that physical health problems were directly diagnosed by a physician and not reported by patients. Past-year migraine was found to be associated with depression, dysthymia, bipolar disorder, panic attacks, panic disorder, agoraphobia and simple phobia.

Interestingly, a recent study investigated the presence of migraine with and without aura, probable migraine and no migraine, according to the IHS criteria, in a large sample of concordantly depressed sibling pairs [29]. The findings supported the hypothesis that the two forms of migraine are different, migraine with aura representing the extreme end of a continuum of migraine familial liability, even in a defined psychiatric population (i.e. depressed patients). Accordingly, in a case–control study, it was recently observed that not only there is a general relationship between recurrent headache and depression, but also a specific association between depression and migraine with aura [30].

## Studies on clinical samples

Since the introduction of the IHS criteria (1988) [14], several investigations have been carried over in clinic-drawn samples, with the aim of comparing migraine with other forms of headache (tension type headache, chronic daily headache, transformed migraine, and daily headaches with chronic substance abuse). None of the studies comparing migraine and tension-type headache could find significant differences in terms of psychiatric comorbidity [31–33], whereas the risk of psychiatric disorders was found to be increased in chronic headache, and particularly transformed migraine, as compared to episodic migraine patients [33–36]. In particular, a study by our group [34] investigated comorbidity with anxiety and depression in several groups of patients, including low back pain patients. While extrapolations of findings should be done cautiously (due to the fact that patients suffering from migraine with interval headache and patients with chronic tension type headache were considered as a whole sample), a significant comorbidity was observed in all groups of patients. Comorbidity was even more pronounced in patients who had had chronic headache for more than 5 years than in those with shorter disease duration. Interestingly, in this study all psychiatric disorders except for somatoform disorders were found to be associated with headache, suggesting that psychiatric comorbidity may be confined and specific to headache category, and it is not merely accounted for by the coexistence of chronic pain. Another study [33] compared migraine sufferers with daily headache patients with chronic substance abuse, and found that major depression was twice as frequent in patients with analgesic abuse. Similarly, other authors [35] demonstrated an increased risk of major depression, panic disorder and social phobia in patients with transformed migraine and chronic substance use, even after adjustment for age and gender. Juang et al. [36] compared patients suffering from transformed migraine with those suffering from chronic tension-type headache, and found a higher frequency of anxiety in transformed migraine patients after adjustment for age and gender. However, similar to the other studies in the field, the role of the diagnostic criteria for substance abuse or dependence criteria was not critically considered. Other authors [37] found that patients with migraine show higher severity of somatic, depressive and anxiety complaints. In addition, migraine appeared to be the strongest independent factor in predicting somatic severity of major depressive disorder, even after controlling for anxiety comorbidities and demographic variables. Recently, in a cross-sectional study panic disorder was found to prevail in migraine compared with tension-type headache or migraine plus tension-type headache, and the association was stronger when migraine was compared to pure tension-type headache; similarly, obsessive–complulsive disorder was more closely associated with migraine than to tension-type headache [38]. The importance of the impact of psychiatric comorbidity on chronicity and impaired quality of life in chronic daily headache sufferers has been pointed out by a recent selective overview [39]. In particular, the complex interplay of factors underlying the relationship between migraine, suicide risk and mood disorders deserve scientific interest and better methododologically based investigation [40].

## From children to grown-up

The hypothesis of a relationship between migraine and psychiatric disorders beginning with anxiety in childhood and adolescence, followed by migraine and later depression [16, 20, 26, 27] pointed the attention on the role of pediatric age.

As previously reported, the presence of psychiatric disorders is more related to severity and frequency of non-migrainous headache than to migraine [24]. The higher prevalence of comorbid psychiatric disorders in chronic daily headache than other headache subtypes both in children/adolescents and adults further supports this finding [41]. The burden of childhood adversities on chronic daily headache did not differ between chronic migraine and chronic tension-type headache in a population study [11].

It has been hypothesized that chronic illness in general, rather than a specific disorder, explains variations in psychological functioning between chronically ill and healthy children. This to stress that psychiatric disorders may not specifically relate to migraine per se, but to migraine as a kind of disabling and recurrent pain. Cunningham [42], comparing migraine and chronic non-headache pain samples, found no difference in anxiety and depression levels between the two groups with chronic pain, with respect to pain-free controls. It is noteworthy that studies looking for differences between migraine and other headache subtypes did not find specific psychological characteristics between migraineurs and tension-type headache sufferers [32, 43]. Recently, a study comparing headache patients and patients with recurrent abdominal pain did not find differences by the psychological point of view (internalizing vs. externalizing disorders) [44]. This suggests that in pediatric age the role of psychological factors might be more related to the frequency and severity of headaches, than to sole migraine. Recently, a population based study on 13–15 year adolescents found an increased risk of suicidal ideation among those with migraine with aura and high frequency non-migrainous headache; the risk increased with increasing frequency of attacks [25].

From the psychiatric point of view, studies consider often “headaches” and/or “abdominal pain” as somatic complaints that are symptoms of child or adolescent psychopathology. Livingston et al. [45] found that between 25 and 30% of children admitted to a psychiatric hospital had physical symptoms, including headache, food intolerance, abdominal pain, nausea and dizziness. Different interpretations have been suggested by other studies. Andrasik et al. [46] found a greater number of somatic complaints in migraineurs and higher ratings of depression and anxiety among migrainous adolescents, compared to matched headache-free subjects. The suggested hypothesis was that “frequent, unexplainable and intense head pain would likely lead to heightened levels of depression and anxiety”. Another aspect to be borne in mind is the presence of subclinical conditions, such as in patients without defined psychopathology but with psychological distress following life-events (e.g. parental divorce) or personality characteristics (e.g. tendency to perfectionism), aspects that may contribute to trigger headache. The diagnostic workup of headache in children and adolescents should always include a psychological assessment for a complete framing of the clinical condition. A recent clinical study [47] showed a connection between childhood maltreatments, adult chronic and/or severe migraine and major depression: migraineurs with current depression reported more frequent physical and sexual abuse compared to those without depression; women with major depression were more likely to report sexual abuse occurring before 12 years, and the relationship was stronger when abuse occurred both before and after 12 years; women with migraine and depression were four times more likely to have a history of some type of childhood maltreatment. These findings outline the interface existing between neurology and psychiatry, organic and psychological, and likely genetic and environmental factors linked to migraine.

A study in a psychiatric setting [48] also showed that headache is the most frequent somatic symptom in children and adolescents referred for emotional and behavioural disorders, as well as in patients with depression and/or anxiety. With regard to gender differences, females appear to be more affected. A population, prospective cohort study on headache adolescents [49] showed gender differences in comorbid associations, with allergy, bronchial asthma, diabetes mellitus and stomach-ache more common in boys, and psychiatric symptoms and sleep disturbances in girls. A 1-year longitudinal study in adolescent headache sufferers [50] found depression, insomnia and low self-esteem associated to headache occurrence, and a likely temporal trend was suggested, with depression and low self-esteem preceding headache onset, even if only in girls. However, the study did not apply ICHD criteria and other standardized (DSM-IV or ICD-10) diagnostic criteria (e.g. it was unclear whether or not low self-esteem or insomnia were related to depression itself). A more recent study [51] showed an increased risk for females of reporting higher levels of depression (and anxiety), and an elevated risk of developing chronic daily headache and medication overuse when patients showed psychiatric disorders as well. The negative effect of the presence of psychiatric disorders in general and depression in particular on the outcome is not new in literature [41, 52], and was recently confirmed in a population based study, including a 2-year follow-up [53].

Similarly to migraine, psychiatric disorders run in families [20]. Anxiety and mood disorders are particularly frequent in migraineurs and their relatives [20]. A recent study [53] showed that psychiatric disorders are equally represented in migraine and other headache subtypes. Noteworthy, parents of migraine children showed a significant higher comorbidity with psychiatric disorders than parents of children with other headache subtypes. This aspect requires attention, because it is the first point clearly differentiating migraineurs from other headache patients, even if further studies are required to support this finding. Migraine children seem to be characterized by a higher prevalence of headache familial recurrence and psychiatric disorders in parents, than other headache subtypes [54]. Both psychiatric comorbidity and headache familial recurrence are also very frequent in children with other headaches, but they can occur together or alone. This pattern of results suggests that anxiety/depression and headache familial recurrence act as additive factors in non-migrainous headaches, while in migraine they might represent, together with psychiatric disorders in parents, interrelated aspects of a more complex relationship. This means that the higher the weight of headache familial recurrence the higher the possibility that children show psychiatric comorbidity, but only in the case of migraine.

In most part of the studies in children and adolescents with migraine, the association with anxiety (and mood) disorders appears to be an important topic, even though a recent systematic review suggested overall inconclusive evidence on this matter [55]. The presence of depression is associated with a poor outcome for any headache subtype. Females have an increased risk for any of the above items. Whether the association is specifically linked to recurrent headache or to headache as a chronic pain is unclear. Moreover, we do not know exactly what happens in children, because depression in children has specific clinical symptoms that complicate its recognition and diagnosis, so that studies might have underreported or misdiagnosed it. The aetiology of the comorbid association is also unclear, and there are no studies on pharmacological and/or non-pharmacological treatment of the comorbid disorders due to the young age. Like in adults, further studies based on proper assessment tools and fulfilling the international systems of classification of both headache and mood disorders are required.

## Metanalysis of studies investigating the association of migraine and depression

With particular regard to the most investigated psychiatric comorbidity, i.e. depression, the crucial issue is whether depression is more frequent in individuals with headache and particularly with migraine and vice versa. An answer to this question may arise from studies that investigated the occurrence of these two disorders in the same population. The most reliable design of study to provide evidence on the association of the two diseases and on its nature is the cohort study. This study method consists of the analysis of the occurrence during time of depression in person with headache without depression and in normal non-depressed populations. The strategy for investigating the relationship between the two diseases may obviously be reversed looking for the occurrence of headache in depressed persons without headache. Less convincing evidence on the existence of an association between the two diseases is derived from cross-sectional studies where the temporal sequence of occurrence, and possible cause–effect relationship, are more hardly recognized. Another study design for investigating the association of two diseases is the case–control study. In case–control studies, a group of subjects with the disease of interest (cases) is compared with a group of subjects without the disease of interest (controls). This type of study needs accurate study designs in order to control for possible bias due to the effect of confounders.

Using as key words headache or migraine and depression, we retrieved in medline 47 studies on the issue of the relationship between headache and depression. Most are cross-sectional, a minority are case–control studies; no cohort studies have been retrieved. Three criteria have been used in the selection of the papers for the metanalysis. The first was the consistence of the paper content with the issue of interest. The second was the presentation of original data. The third was the possibility of deriving crude data from the paper, and this was a *conditio sine qua non* for the execution of the metanalytic procedure. Of the 47 studies, 17 were inconsistent with the issue of the relationship between migraine and depression; 5 were reviews or editorials presenting no original data; finally, for 13 studies it was impossible to derive the crude data. 12 studies remained therefore available for the metanalysis (Table 1) [7, 19, 23–25, 27, 56–60].

**Table 1:** Prevalences and odds ratios of depression in migraineurs with respect to subjects without migraine for each one of the 12 considered studies

Study	Subject age range (years)	Diagnostic tool	Without migraine Depression		With migraine Depression		OR (95% CI)
			No	Yes (%)	No	Yes (%)	
Ratcliffe et al. [19]	18–65	CIDI	3,762	305 (7.5)	455	79 (14.8)	2.1 (2.1–1.7)
Hung et al. [37]	ND	HAMD–S DSSS	62	20 (24.4)	38	35 (47.9)	2.8 (2.9–1.5)
Jette et al. [18]	15–over 65	CIDI	31,772	1,122 (3.4)	3,641	343 (8.6)	1.8 (1.8–1.6)
Camarda et al. [23]	ND	CES-D	1,043	242 (18.8)	80	71 (47.0)	3.8 (3.8–2.7)
Merikangas et al. [27]	27–28	SPIKE	367	29 (7.3)	52	9 (14.7)	2.2 (1.0–4.8)
Breslau et al. [56]	25–55	CIDI	453	86 (16.0)	287	209 (42.1)	3.8 (2.9–5.1)
Samaan et al. [30]	19–85	SCAN, BDI	808	1,070 (57.0)	43	189 (81.5)	3.3 (2.4–4.6)
Lipton et al. [21]	18–65	PRIME-MD	315	64 (16.9)	206	183 (47)	4.4 (3.2–6.0)
Lanteri-Minet et al. [58]	ND	HADS	6,651	1,264 (15.7)	1,465	442 (23.2)	1.6 (1.4–1.8)
Breslau et al. [24]	25–55	CIDI	492	94 (16.0)	318	218 (40.7)	3.6 (2.7–4.7)
Kececi et al. [59]	Over 18	DSM-IV	682	102 (13.0)	110	53 (32.5)	3.2 (2.2–4.7)
McWilliams et al. [60]	25–74	CIDI-SF	1,319	185 (18.5)	243	97 (28.5)	2.8 (2.2–3.7)
							**2.2 (2.0–2.3)**

*HAMDS* Somatic items of the Hamilton Depression Rating Scale, *DSSS* Depression and Somatic Symptoms Scale, *CES*-*D* Center for Epidemiologic Studies Depression Scale, *HADS* Hospital Anxiety and Depression Scale, *SCAN* Schedule for Clinical Assessment in Neuropsychiatry, *BDI* Beck Depression Inventory, *CIDI (SF)* Composite International Diagnostic Interview (Short Form), *SPIKE* Structured Psychopathological Interview and Rating of the Social Consequences for Epidemiology, *PRIME*-*MD* Primary Care Evaluation of Mental Disorders

Table 1 shows the prevalences and the odds ratios of depression in patients with migraine with respect to subjects without migraine for each one of the 12 considered studies. Across the studies, the prevalence estimates of depression were highly variable, whereas the ratio of depression prevalence between subjects with and without migraine was more consistent. The prevalence estimates varied from a minimum of 3.4% to a maximum of 24.4% in individuals without migraine. The corresponding figures among migraineurs were 8.6 and 47.9%. The individual study odd ratios had a minimum of 1.8 and a maximum of 4.4; however, the confidence limits of each study were comprehensive of almost all the point estimates of the other studies. The overall risk estimate gave an odd of depression for people with migraine with respect to people without migraine of 2.2, with a 95% confidence interval of 2.0–2.3. It should be noted that the two largest studies [18, 56] reported the lowest odd ratios (1.6, 1.8, respectively). Furthermore, these two studies were characterized by high number of participants and this may have influenced the results and decreasing the mean values of the overall studies (Table 1).

In conclusion, all the individual studies and the overall metanalytic investigation show that depression is almost two time more frequent in subjects with migraine than in people unaffected by headache.

## Mechanisms of migraine psychiatric comorbidity

Comorbidity between migraine and psychiatric disorders has been extensively studied, but the mechanisms underlying this phenomenon are far from clear. Direct and indirect data mainly stem from longitudinal studies investigating the order of onset of each condition, the changes in severity/evolution of one disorder if another is present, and the co-transmission of these disorders within families. The possible mechanisms of comorbidity are several [61]. The association of two disorders may be a result of chance. One disorder may cause another disorder (such as diabetes causes diabetic neuropathy). Shared environmental risk factors may underlie both disorders (such as head trauma causing both post-traumatic headache and post-traumatic seizures). Finally, genetic or environmental risk factors may produce a brain state resulting in both conditions. In the latter case, common neurobiological determinants may account for both disorders, and this mechanism appears to be the most appropriate for comorbidity of migraine and depression. Following this conceptual view, the evidence from literature [6, 60, 61] thus points to three main potential mechanisms, as follows:

1. psychiatric disorders are causal factors in the development of migraine. In this case, psychiatric disturbances are responsible for a full expression of migraine, and under particular circumstances for the evolution of migraine in a daily pattern (chronic migraine)
2. migraine is a causal factor in the development of psychiatric disorders. In this case, the repetition of intense and/or long lasting pain episodes may facilitate the development of anticipatory anxiety and/or depression
3. shared aetiological factors and common determinants explain the co-occurrence of both entities. In this case, there is no clear causal association, and a common substrate (e.g., deranged activity of neurotransmitters or receptors) may cause both migraine and the comorbid psychiatric disorder.

With particular regard to the relationship between the frequency of psychiatric comorbidity and the severity of migraine, some evidence [33] suggested that there is a significant association between frequency and duration of the attacks, but not with the intensity of pain. A correlation was subsequently observed between the evolution of headache and the presence of anxiety or depression. In this respect, several studies have been focused about onset of specific disorders. In one study [28], anxiety was shown to precede migraine in most patients, which in turn preceded depression. These findings were very similar to those obtained by other authors [17, 27], indicating that the onset of anxiety preceded that of migraine, which in turn preceded that of depression in most patients, and that the ages of onset of each disorder were significantly correlated.

With the aim of elucidating the role of psychiatric disorders as possible risk factors for the onset of migraine, some authors [17] showed that only the anamnestic presence of phobic disorder was predictive of the onset of migraine, at variance with that of affective disorders. The latter observation is consistent with other reports [17, 27, 28], strongly suggesting that depression and dysthymia are not risk factors for the onset of migraine.

Other studies have investigated the reciprocal relationship of migraine and psychiatric disorders. The risk of onset of depression or panic disorder during a follow-up period of over 1 year was found to be slightly greater in subjects with a history of migraine (15.5%) than in subjects with current migraine (13%) [28]. Recently, using a complex statistical hazard model, the same authors [24, 63] found no preferential order of onset for depression or panic relative to migraine, although a trend towards an order of onset of major depressive episodes in relation to severe non-migraine headache was observed. All these findings thus support the view that the comorbid disorders are bidirectionally linked. In this regard, a follow-up study in children showed that anxiety predicted the persistence of headache in both migraine and tension-type headache patients [32].

It would therefore appear that only phobic disorders predict the onset of migraine, and that a clear bidirectional relationship exists between migraine and depression or panic disorder, that is, each disorder may represent a risk factor of the other.

Comorbidity can be alternatively explained by the hypothesis that common genetic and/or environmental risk factors may underlie both migraine and psychiatric disorders. One of the earliest reports [65], was inconclusive in this respect, though IHS criteria were not yet available. By duly applying IHS criteria, it was later found that there was indeed no familial crosstransmission between migraine and affective or anxiety disorders [20]. In another report, the risk of bipolar disorder was not increased in the relatives of non-bipolar migraine patients [66]. Taken together, these studies did not support the view that depressive and bipolar disorders share common genetic determinants with migraine. However, everyday experience indicates that in both migraine and affective disorders the frequency of episodes can increase with time, and that both disorders can progress to more chronic states with poor recovery between episodes and development of drug resistance. This suggests that sensitization phenomena may underlie both disorders [67].

Biologically based studies have also tried to address the issue of migraine psychiatric comorbidity. An association was reported between a particular dopamine D2 receptor genotype and comorbid migraine with aura, major depression and generalized anxiety disorder [68]. A lifetime history of major depression was reported to be associated with reduced tyramine conjugation (a marker of endogenous depression) in migraine patients compared with controls [69]. This observation argues against the hypothesis that depression may develop as a psychological reaction to migraine attacks. Serotonin receptors and transporters, and catecholamines have also been implicated in migraine as well as various psychiatric disorders [5, 70], and there is evidence supporting the effectiveness of several antidepressants (including SSRI abd SNRI) in the prevention or treatment of migraine [71]. Female migraineurs often experience attacks associated with falling estrogen levels around menses, and mood disturbances in women often coincide with menses, as well as the postpartum period and the perimenopausal period. Ovarian hormones, modulating numerous neurotransmitters, therefore appear to play an important role in migraine as well as in depression [72]. However, the neurobiological mechanisms of migraine psychiatric comorbidity are still far from being clear.

## Impact of psychiatric comorbidity on migraine

Comorbidity with psychiatric disorders raises the global burden of migraine. Increasing evidence suggests that migraine in comorbidity with psychiatric disorders is associated with poorer health-related outcomes [18]. Several studies have so far examined health-related outcomes of migraine, investigating variables such as disability, restriction of activity, quality-of-life or mental health care utilization [73–76]. In these cases, however, investigation was regularly restricted to migraine, without taking into consideration any possible psychiatric comorbidities. When comorbidity was taken into account, in patients suffering from both conditions the prevalence of disability, restriction of activities, poorer quality of life and mental health care use was found to be higher than in those with only one of the two conditions, and even higher than in those with neither condition [18]. Other authors [77] reported that male patients with comorbid bipolar disorder and migraine were more likely than those without migraine to utilize mental health care services. The same group found that bipolar females with comorbid migraine were more likely to require assistance in their daily routine when compared with bipolar females without migraine. Patients with migraine were found more likely to have a history of various psychiatric disorders and concomitantly to report job absenteeism, to rate their general health as fair or poor, and to use mental health services [28]. Recent evidence from large populations of patients has confirmed that single-item scales are valid and reliable to assess symptom severity, psychosocial function, and quality of life [78]. Health-related quality of life was reported to be generally lower in patients with comorbid migraine and one mental health disorder [21, 63, 74]. Similarly, in patients with MDD, the coexistence of migraine was shown to predict a significant negative impact on all physical subscales and vitality in the assessment of quality of life [79]. The same group reported that subjects with migraine, anxiety, or chronic depression had higher depression scores and poor quality of life; in addition migraine, specific phobia, and panic disorder were important and independent comorbidities predicting quality of life [80]. The presence of migraine should therefore be considered as an important clinical symptom in all clinic-based samples of depressed patients. However, as already pointed out [18] the currently available studies does not elucidate whether health-related outcome variables are specific to migraine or to mental disorders [18]. Different mechanisms may link migraine, psychiatric disturbances and poor quality of life. In patients with migraine and comorbid psychiatric problems, the impairment in quality of life may indeed mirror a real ill condition, or an altered perception of life circumstances, or both. Prospective studies will probably help to clarify these important points.

## Conclusions

Though not easily comparable due to differences in methodology to reach diagnosis (i.e. psychiatric interviews and scales), population based studies generally indicate an increased risk of affective and anxiety disorders in patients with migraine, compared to non-migrainous subjects. There would also be a trend towards an association of migraine with bipolar disorder. By contrast, there is definitely no comorbidity with substance abuse/dependence. With respect to migraine subtypes, comorbidity (e.g. suicide attempts, bipolar disorder) mainly involves migraine with aura rather than the form without aura.

However, the lack of diagnostic recognition of certain forms of migraine, such as chronic migraine, due to the use of the first version of IHS criteria, may have significantly affected the results of several studies. Another limitation is that some of these studies were carried over within psychiatric research protocols, and thus were not originally designed to investigate the comorbidity between migraine and psychiatric disorders.

Apparently, no significant difference exists between migraine and tension-type headache patients in terms of prevalence of psychiatric comorbidity. By contrast, patients suffering from migraine show a decreased risk of developing affective and anxiety disorders compared to patients with chronic daily headache. It would also appear that affective and anxiety disorders prevail in patients with chronic forms of headache and substance use than in patients with migraine alone. Furthermore, patients with “transformed” (or chronic) migraine show an increased prevalence of affective and anxiety disorders compared to patients with simple migraine or chronic tension-type headache. Although early studies suggested that there is a correlation between frequency of headache and frequency of anxiety or depressive disorders, little evidence support a correlation between the severity of migraine and anxious or depressive symptoms.

In conclusion, the mechanisms underlying migraine psychiatric comorbidity are presently poorly understood, but issues concerning this topic remain a priority for future research. Psychiatric comorbidity indeed affects migraine evolution, may lead to chronic substance use and may change treatment strategies, eventually modifying the outcome of this important disorder.
